# mHealth Technology Use and Implications in Historically Underserved and Minority Populations in the United States: Systematic Literature Review

**DOI:** 10.2196/mhealth.8383

**Published:** 2018-06-18

**Authors:** Charkarra Anderson-Lewis, Gabrielle Darville, Rebeccah Eve Mercado, Savannah Howell, Samantha Di Maggio

**Affiliations:** ^1^ Department of Public Health University of Southern Mississippi Hattiesburg, MS United States; ^2^ College of Public Health University of Georgia Athens, GA United States; ^3^ College of Medicine Department of Pediatrics University of Florida Gainesville, FL United States

**Keywords:** mHealth, mobile health, digital health, smartphone, text messaging, minority, ethnic group, health disparity, underserved population

## Abstract

**Background:**

The proportion of people in the United States who are members of at least two ethnic groups is projected to increase to 10% by the year 2050. This makes addressing health disparities and health inequities in minority populations increasingly more difficult. Minority populations, including those who classify themselves as African American and Hispanic, are using mobile phones to access health information via the internet more frequently than those who classify themselves as white, providing unique opportunities for those in public health and health education to reach these traditionally underserved populations using mobile health (mHealth) interventions.

**Objective:**

The objective of this review was to assess studies conducted in the United States that have used mHealth tools and strategies to develop and implement interventions in underserved populations. This review also examines the ways in which mHealth strategies are being employed in public health interventions to these priority population groups, as mobile phone capabilities include text messaging, mobile apps, internet access, emails, video streaming, social media, instant messaging, and more.

**Methods:**

A systematic literature review was conducted using key search phrases, the matrix method, and Preferred Reporting Items for Systematic Reviews and Meta-Analyses flowchart diagram to identify key studies conducted between the years of 2009-2016 in the United States. These studies were reviewed for their use of mHealth interventions in historically underserved and minority populations.

**Results:**

A total of 16,270 articles were initially identified using key search phrases in three databases. Titles were reviewed and articles not meeting criteria were excluded, leaving 156 articles for further review. After additional review for relevance and inclusion criteria, 16 articles were qualified and analyzed.

**Conclusions:**

mHealth is a promising area of development for public health and health education. While successful research has been done using text messaging (short message service, SMS) and other mHealth strategies, there is a need for more research using mobile phones and tablet applications. This literature review demonstrates mHealth technology has the ability to increase prevention and health education in health disparate communities and concludes that more specified research is needed.

## Introduction

### mHealth in the United States

Mobile phone use has become a part of daily life for most people in United States with wireless users sending and receiving an average of 6 billion text messages per day or 69,635 text messages every second [1,2]. Mobile cellular subscriptions have reached 90% of the world’s population and have reached nearly 80% of the global population living in rural areas [3]. Serving as a 2-way interactive communication tool, mobile health (mHealth) can present opportunities for researchers and program developers to capitalize on the existing cultural behaviors of their target populations, given their rates of access to and use of mobile technology [4]. As van Velthoven et al explain, “the widespread use of mobile devices and almost universal coverage of the world’s population by a network signal has given mHealth a great opportunity to improve global health” [5]. As a result, mHealth interventions have become increasingly common in low-income and third world countries. In areas where financial resources and health care workforces are lacking, and high rates of disease occur, short message service (SMS) text messaging could potentially make the ability to obtain to health care more accessible and more affordable [6].

With almost 100% of the total US population owning a mobile phone of some kind, health communication technologies could be incredibly beneficial for those with limited English proficiency, individuals with disabilities, and persons living in rural and other isolated geographic areas [7]. The ability to tailor mHealth interventions in a culturally competent manner and implement program curriculum at the literacy levels of the target population makes mHealth not only adaptable but also optimal. The mobility of the technological platform allows population groups experiencing the greatest health disparities to have improved access to health care and health resources.

Mobile phones are a more cost-effective way to access health information for those of a lower socioeconomic status (SES). In addition, mobile technologies have potential to ameliorate the management of chronic diseases and smoking cessation while simultaneously improving communication between patient and provider [8]. A recent report by the Institute of Medicine notes, “[health information technology] provides an opportunity for engaging populations not historically well served by the traditional health community…The impact of facilitating patient and population contribution to, and control of, their health information has the potential to provide further insights into, and opportunities to address, disparities in underserved populations” [9,10].

As the US population diversifies, health disparity incidence and prevalence rates are not expected to decline; health disparities are predicted to be even more difficult to diagnose. According to Yancey et al, the proportion of people in the United States who are members of at least 2 ethnic groups will increase 10% by the year 2050, complicating assessments of health disparities [11]. However, recent technology trends in the United States indicate mobile phone usage and smartphone adoption rates by those experiencing the highest rates of health disparities are increasing, providing a means for those who work in public health and health education to reach these populations through mHealth interventions. As of January 2017, technology trends indicate that 95% of American adults own a mobile phone, 77% own a smartphone, 22% own an e-reader, 51% own a tablet computer, and 78% own a desktop or laptop computer [7]. The objective of this review was to assess studies conducted in the United States that have used mHealth tools and strategies to develop and implement interventions in underserved populations. This review also examines the ways in which mHealth strategies are being employed in public health interventions to these priority population groups, as mobile phone capabilities include text messaging, mobile apps, internet access, emails, video streaming, social media, instant messaging, and more.

### mHealth: Text Messaging

The use of SMS text messaging and mobile apps for improving health and providing health information [12] are gaining momentum for incorporation into interventions and programmatic efforts in the United States. Statistics regarding SMS text message trends in the United States indicate 80% of all US mobile phone owners text, 92% of US smartphone owners text, and US SMS users text, on an average, 35 texts per day [13]. SMS text messaging interventions in health education and behavior show promise in underserved and minority populations due to increasing usage trends. To date, research shows, African American and Hispanic mobile phone users are more intense and frequent users as compared to white mobile phone users. As of 2011, non-white mobile phone users (African American users in particular) were found to text more often than white mobile phone users, and those with lower levels of income and education were found to text more often than those with higher levels of income and education. Smartphone owners (mean 52.0; median 20) also send and receive a significantly larger number of texts per day than owners of more basic phones (mean 29.7; median 10) [14]. Because more than 70% of all African American and English-speaking Latino mobile phone users use text messaging, versus just over half of white mobile phone users, and 92% of black adults are mobile phone owners, and 56% own a smartphone of some kind [13], the usage of mHealth strategies as a tool to eliminate health disparities shows opportunities of promise.

### mHealth: Phone Internet Use Trends

The utilization of mobile phones for internet access allows for a higher likelihood of mHealth interventions and programs being used among underserved populations. Referencing internet usage, approximately 88% of Americans (9 out of 10 individuals) use the internet on a consistent basis and 73% use high speed broadband service in their home. Among all Americans, those who classify themselves as Hispanic (58%) and African American (65%) have the lowest rates of high-speed internet access at home compared those who classify themselves as white [7]. This has significantly increased from projections made by Pew Research Center in 2000 and 2010, indicating a doubling of internet use from 11% to 21% [13]. These findings are of particular interest to those interested in trends related to young people, and those who classify themselves as Latino or African American, as these groups are significantly more likely than other groups to have mobile internet access [13].

Not only has there been an increase in minority internet use overall, but the use of mobile phones to access the internet has also increased bridging the digital divide between upper and lower classes [15]. A 2013 report indicated that those who classify themselves as Latino or African American are more likely to use a mobile phone to search for health information via the internet [16]. This is reiterated by 2016 data which shows that those who classify themselves as Hispanic (23%) or black (15%) tend to be more smartphone dependent and are smart-phone only internet users, meaning that they only utilize their smartphone to access Web-based information [7]. In fact, some innovative uses of mHealth technologies have been successfully implemented among low-income pregnant women, Spanish-speaking migrant workers, and homeless and at-risk youth [17,18]. This, then, validates the importance of examining these trends.

## Methods

### Overview

Keywords were searched using the institution’s e-library database to access peer-reviewed journal articles from a variety of electronic databases and Web-based academic journals including PubMed, Health Reference Center, SAGE Journals, and Info Trac. mHealth is a newer concept in the field of public health and technology; therefore, only research literature published in English from 1 February, 2009 to 2 February 2016 was considered and included. The year 2009 was selected because in the United States, mobile phones started to evolve from the basic mobile phone to more of a smartphone [19]. Articles also had to be scholarly publications with full text available on the Web for review. The key search terms consisted of 2-3 components, a word that described historically underserved or minority populations, a word that described mHealth technology and related technology, and a word or phrase that described health, public health, or health access. Keywords related to historically underserved and minority populations, health, and mHealth technology were used to create a total of 18 preselected search phrases. The complete list is available in Textbox 1.

### Selection Criteria

Before conducting the review, the inclusion and exclusion criteria were established. To be included, articles had to (1) focus on a specific priority population group or subgroup; (2) discuss technology use, specifically mHealth among priority population; and (3) relate to public health, health education programs, and health behavior interventions in the priority populations. To ensure that the third criterion was met, search terms were filtered by subject terms which included (1) medicine and public health; (2) health care; (3) public, environmental, and occupational health; (4) health; (5) health aspects; and (6) public health.

Studies were excluded from initial review if they were (1) not related to health, (2) addressed research conducted outside the United States, (3) discussed the historically underserved population as current or future employees of the health system instead of as patients, (4) surveyed members of the underserved population on their ownership and usage of mobile phones but did not include an intervention, or (5) surveyed members on their openness to an intervention utilizing mHealth strategies but did not implement an intervention aimed at improving health outcomes. In addition, articles were excluded if manuscripts were not written in English or examined cost as the main variable.

### Data Extraction and Coding

Using the Preferred Reporting Items for Systematic Reviews and Meta-Analyses (PRISMA) Flow Diagram as a guide, the systematic literature review results were organized and reported based on identification, screening, eligibility, and inclusion criteria. The matrix method was also utilized for papers that met the inclusion criteria following review of the abstract and full papers. To ensure a systematic method for reviewing and extracting data from the paper, a coding matrix was created with rows and columns summarizing key sections in each article. These included the author, title, journal, year, purpose, study design, theoretical constructs, demographic characteristics of the study participants (to include gender, race and/or ethnicity, and urban and/or rural setting), and how technology was used focusing on which health issue was addressed.

**Search strategy terms and phrases for the systematic literature review.**

Search term and phrase:

1. Short message service (SMS) health, minority groups
2. mHealth, health disparities
3. Mobile health, ethnic groups
4. Internet, racial health
5. Text message, health, underserved populations
6. SMS, cultural diversity, health
7. Health technology, health education, underserved populations
8. Digital health, diversity, health education
9. Mobile phone, ethnic groups, public health
10. Smartphone, health, ethnic groups
11. Tablet, health equity, underserved populations
12. Health information technology, cultural competence, ethnic groups
13. mHealth, underserved populations
14. Mobile apps, cultural competence, minority health
15. Health communication, health equity, ethnic groups
16. Diversity, health technology, minority group
17. Health literacy, health disparities, mobile health
18. Health equity, educational technology, health disparities

## Results

### Principal Results

**Figure 1:**
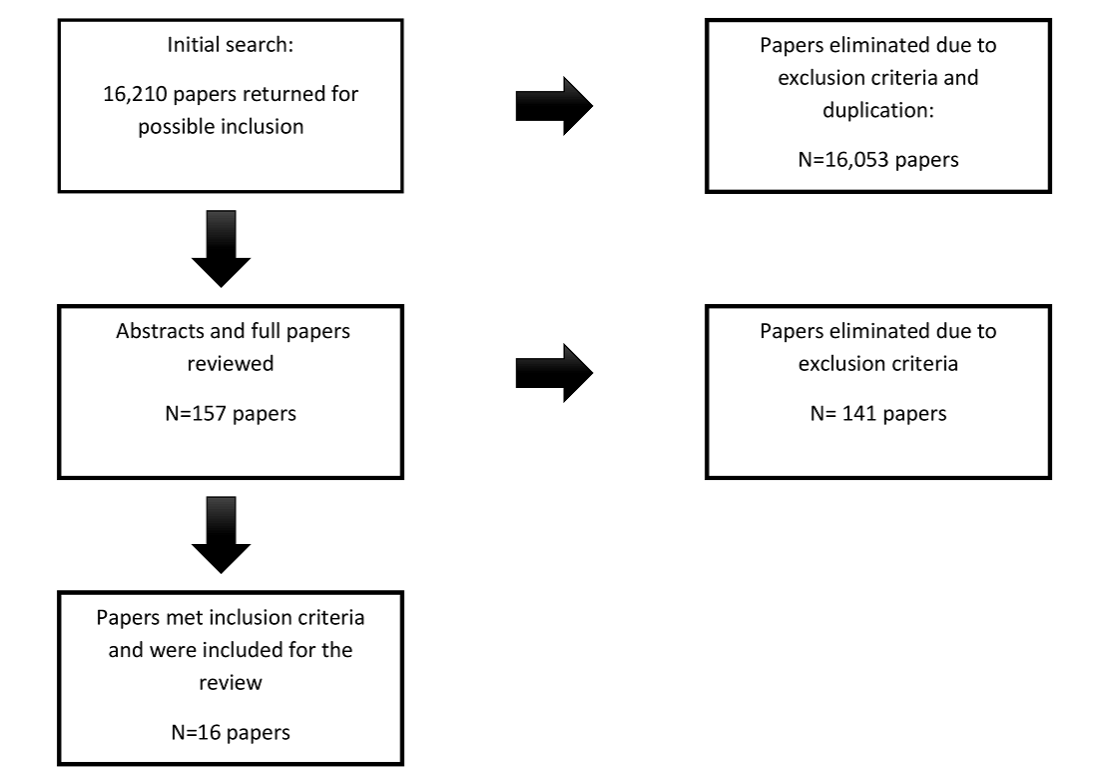
Preferred Reporting Items for Systematic Reviews and Meta-Analyses (PRISMA) literature search results.

The 18 search phrases returned 16,210 articles; however, 16,053 articles were excluded either because they were duplicates or because they did not meet initial inclusion criteria based on their titles. Abstracts and full papers were pulled and reviewed (N=157), and an additional 141 articles were excluded based on the inclusion criteria stated above. A total of 16 papers met the inclusion criteria specifications and were included for review, as shown in Figure 1.

Of the 16 articles reviewed, all were published during the years 2010-2016. The most prevalent demographic variables included being a female (woman), urban and/or metropolitan setting, and having participants aged between 15 and 30 years. One study addressed health behaviors in men, and another’s target population consisted of children and adolescents in which parents acted as mediators for change due to technology access [20,21]. Moreover, 9 of the 16 studies focused on populations who classify themselves as African American or a mix of populations who classify themselves as African American and Latino and/or Hispanic, and 7 studies targeted predominately Hispanic and Latino populations. Finally, 1 study addressed Korean American women.

The 2 most commonly included issues were diabetes and sexual, reproductive, and maternal and child health. Other key issues also included influenza vaccinations and health and HIV and AIDS prevention for high-risk population groups [21]. Each study identified additional characteristics that determined participant eligibility. These are presented in Multimedia Appendix 1.

The review also assessed differences relating to the methodology in research design for the mHealth interventions. Only 7 of the 16 studies had a theoretical grounding or based their research design and evaluation on theoretical constructs. Most of the studies were quantitative rather than qualitative; however, 1 did not employ either methodology. Moreover, 5 of the studies were randomized controlled trials and reported 2 statistically significant outcomes, 5 were pilot studies, and 1 was a prospective cohort study. The 5 remaining studies had mixed designs.

Although all studies utilized mHealth technology in the implementation of their intervention, the method varied from study to study. In 3 of the studies, mobile phones were provided by researchers or the program coordinator. In the remaining studies, program participants had to have a working mobile phone to be enrolled in the intervention. In 1 intervention designed to provide counseling services to parenting teen mothers, phones were contracted and limited to a certain minute limit per month, with no texting or internet features [18]. In another study, smartphones provided by researchers were used as an education platform by streaming video episodes to prevent HIV sex risk behaviors [22]. Only 8 of the 16 studies used SMS text messaging as a communication strategy.

### Article Content Overview

#### HIV Prevention: The 411 Safe Text Project

The 411 for Safe Text project is an mHealth intervention that utilized SMS text messages to deliver HIV prevention program among 60 young black men in Philadelphia aged between 16 and 20 years, who met eligibility criteria and had a mobile phone. Moreover, 30 were assigned to the control group and 30 to the intervention group. The men in the study received SMS text messages 3 times a week for 12 weeks about nutrition (control group) and sexual health (intervention group), with follow-up assessments performed after 12 weeks and afterward. This study aimed to test the feasibility of enrollment, participation, and retention of young black men in a SMS text message program for HIV prevention. A total of 67% of control group members completed the assessment and program; 63% of intervention group members completed the assessment and program.

#### Efficacy of a Mobile Phone–Based Counseling Intervention Among Pregnant Teen Mothers

The mHealth intervention conducted by Katz et al recruited pregnant teens aged 15 to 19 years in Washington, DC, to test the efficacy of mobile phone intervention in postponing subsequent teen pregnancies [18]. Comparison was made between the intervention and standard of care to see what effect the intervention might have on the amount of time before the participants experienced a subsequent pregnancy. Intervention group teens received mobile phones for 18 months of counseling sessions, in addition to quarterly group sessions in which minutes, texting, and internet features were limited. Moreover, 249 African American and Latina primiparous pregnant teens aged 15 to 19 years who had not graduated from high school participated in the research study. Within the 2-year follow-up period, 31% of the intervention group and 36% of the usual care group became pregnant again. Although the use of mobile phones allowed for more opportunities to communicate with the participants, inconsistency with counseling sessions over an 18-month period occurred (52% completed). The study presented unforeseen challenges in relation to motivations for participation (free phone was a selling point) and communication.

### Pilot Evaluation of the Text4baby Mobile Health Program

In the study conducted by Evans et al, a pilot evaluation of the *text4baby* mobile health program was randomized and delivered to an intervention group (received usual health care and *text4baby* SMS text messages) and control group (received usual health care) [23]. Specific aims of the text4baby program were to (1) assess exposure, awareness, and reactions to the messages and (2) identify direct effects of the messages on maternal prenatal care and related health attitudes, beliefs, and behavioral outcomes. This program sent text messages that offered immediate just-in-time tips geared at improving prenatal and postpartum health care outcomes in incredibly disparate women. Conclusively, participants sought prenatal care information on the Web (5%), alcoholic consumption decreased after confirmation of their pregnancy (3% to 1%), consumption of 3 or more servings of fruit a day increased (3%), and tobacco use decreased in the last 30 days (6% to 1%). Increased exposure to text4baby messages resulted in program participants being 3 times (odds ratio 2.73) more likely to believe that they were more prepared to be a new mother.

#### Influenza Vaccination in Low-Income Pediatric and Adolescent Populations

In an effort to increase influenza vaccination rates among low-income urban children and adolescents, researchers designed a randomized control trial where parents of the target population would receive SMS text messages promoting influenza vaccines [21]. Conducted in 4 community-based pediatric clinics that primarily serve Latino and publicly insured populations, children and adolescents were eligible for free vaccination through the Vaccines for Children Program. Parents received a series of automated SMS text messages over 5 weeks featuring educational information specific to the child’s age, which provided notification of upcoming vaccine clinics. The messages were personalized based on language preference of the parents and later discontinued once the children or adolescents were vaccinated. As a result of the intervention, a higher proportion of the children and adolescents in the intervention group (44%) received the influenza vaccine compared with the control group (40%).

#### Text4baby Program: An Opportunity to Reach Underserved Pregnant and Postpartum Women

This study was a prospective cohort study conducted in 2 Women, Infants, and Children centers in Atlanta, GA. A total of 468 randomly selected pregnant and postpartum women were asked about their mobile phone use and given information about the text4baby program for enrollment. Pregnant women and new mothers received tailored and targeted SMS text messages on prenatal and postpartum services and behaviors. Conclusively, 9 out of the 10 participants of the text4baby program read the messages and planned on continuing with the program despite disruption of the mobile service. Additionally, underserved pregnant women and new mothers widely accepted the program. Over two-thirds of the women had no month to month mobile phone plan, which affected the response rate and message reception ultimately presenting barriers [17].

#### Streaming Weekly Soap Opera Video Episodes on Smartphones to Reduce HIV Risk Among Black Women

Love, Sex, and Choices is an mHealth intervention that utilized data only on smartphones to stream 12 episodes of a soap opera video series designed to reduce HIV sex risk practices among low SES African American women aged 18 to 29 years. Researchers conducted a randomized control trial assigning 238 participants to both comparison (text) and intervention (video) groups. Researchers were able to track the participant’s trends in actually accessing, watching, and completing the video modules. If participants failed to watch the video by a particular benchmark in the week, reminders were made to account for possible attrition and track intervention fidelity. Individuals in the intervention group were responded to questions about each episode by entering the responses on the phones, whereas the comparison group received only written messages communicating HIV risk reduction on the phones. Regarding the intervention group, 97% of the participants reported that they enjoyed watching the video on the mobile phone, 99% thought it was easily accessible, and 97% thought that using the mobile phone was easy. Also, 95% enjoyed the video and enjoyed being able to watch the videos on their phone wherever they went and 93% believed phone training was very helpful. Advantages to the program included mobility, privacy, and increasing the participant’s self-efficacy smartphone usage. Disadvantages included damaged mobile phones (almost half of 161; n=55), stolen mobile phones (56), misplacing login information, inability to recall passwords, and server issues. An alternative identified to accessing the videos via their emails included the creation of a mobile app. This app provided a new channel to address health disparities in traditionally underserved populations [22].

#### Print Versus Culturally Relevant Facebook and Text Message Intervention to Promote Physical Activity Among African American Women

Joseph et al conducted a research study among African American women looking at the difference between electronic intervention (Facebook and text message) and print intervention (brochures delivered via postal mail) to promote physical activity. Participants (N=29) were randomly assigned to one of the 2 physical activity interventions for 8 consecutive weeks. Moreover, 1 group used Facebook and text message to promote physical activity (F1), and the other group delivered the intervention by mailing brochures to participants’ homes (P1). Participants of the study also self-reported their physical activity levels, psychosocial variables, and satisfaction of the program. Participants in the F1 group showed a decrease in sedentary time and an increase in intensity compared with participants in the P1 group while also demonstrating greater enhancements in self-regulation for physical activity and increased moral support. Satisfaction in this group was high (100%), and ultimately, the F1 group showed more positive outcomes compared with the P1 group [24].

#### Efficacy of a Telephone-Delivered Sexually Transmitted Infection and HIV Prevention Intervention for Adolescents

DiClemente et al performed a randomized clinical trial looking at the efficacy of a telephone-delivered phone intervention for prevention and maintenance program in sexually transmitted diseases and HIV. This particular study used phone counseling to prevent behaviors and sexually transmitted infection (STI) incidence in a 36-month follow-up. Participants were recruited from 3 clinics in Atlanta, GA, including African American adolescent girls aged 14 to 20 years. All participants received primary treatment and were split into 2 groups to receive supplemental treatment to help the effects of the primary treatment. The experimental group (n=343) was provided an STI intervention plan and was contacted by telephone every 8 weeks for 36 months. The purpose of the calls was to reinforce and complement prevention. The condition group (n=359) also received their STI intervention plan, HORIZONS, but they received information that focused on general health. This study looked at 3 behaviors: the proportion of condom-protected sexual acts in 6 months and 90 days before assessments, number of sexual episodes while participants were under the influence of drugs or alcohol, and number of vaginal partners before assessments. Conclusively, fewer participants had chlamydia infections in the experimental group than in the condition group, 20% reduction rate, and also fewer gonococcal infections (48 to 54). Participants in the experimental group had a lower risk for chlamydial infections and reported higher condom use and fewer sexual acts while under the influence of drugs, alchohol or both. STI maintenance interventions in adolescents are critical because the population is at such high risk [25].

#### How Do Mobile Phone Diabetes Programs Drive Behavior Change?

Nundy et al conducted a study to determine how mobile phone programs for diabetes can drive behavior change using a mixed observational cohort study. Participants classified themselves as African American and lived in a working class, urban community. The authors studied the behavioral effects of CareSmart, a theory-driven intervention that includes SMS text messages and remote nurse support. Participants of the study received educational messages and reminders on nutrition, glucose monitoring, medications, foot care, and exercising. On the basis of the participants’ responses to SMS text messages, nurses would contact patients periodically. Of the 372 members that were eligible for the study, 67 participants completed the 6-month program with follow up. One-third of participants reported well-controlled diabetes, one-third reported moderately controlled diabetes, and one-third reported poorly controlled diabetes. At 3 months into the program, researchers conducted a survey that showed improvement in the number of days on which participants were performing self-foot care and exercise compared with the initial start of the program. At 6 months of the program, along with exercising and foot care, healthy eating and glucose monitoring also improved [26].

#### An Automated Telephone Nutrition System for Spanish Speaking Patients With Diabetes

Khanna et al conducted a mobile technology and diabetes study focusing on Spanish-speaking patients. Over the course of a 12-week period, calls were made to participants at least 2 times a week asking participants about food portion consumption and tabling the glycemic index of foods while tailoring messages to each participant based on responses. This study included 75 participants with 1 group receiving an average of 26 calls, 2.2 times per week. An average of 10 phone calls per week were completed. Challenges of the study included difficulty reaching participants and phone numbers becoming disconnected [27].

#### Enhancing a Teen Pregnancy Prevention Program With Text Messaging

Devine et al conducted a pilot study (with a purpose of preventing teen pregnancy via SMS text messaging) that included a total of 96 teens enrolled in a month-long program receiving SMS text messages from Teen Outreach Program Text Plus. The study gathered information on teens’ values, social support, self-efficacy, trouble with the law, and sexual activity. Moreover, 30 males and 29 females, aged 14 to 18 years, were recruited. One-third of the participants were African American and two-thirds were Latino. Before the intervention of the study, focus groups were held with participants to determine how to best implement the SMS text messaging component of the program and concluded that 15% of the participants did not own a mobile phone. The participants that did own a mobile phone stated they were heavy texters. Participants suggested that the 15% of teens without phones should pair up with the ones who did, and also to make it fun by adding games, contests, and fun facts among the Teen Outreach Program group texts. At the start of the program, multiple risky behaviors, such as having sex at an early age, were recorded, with a total of 63% reported having sex at the age of 13 years or younger. Teens that reported an intention to have sex stated that they had no intention of using contraception (fewer than 44%). Conclusively, a total of 23.7% of the teens reported to having more than one partner within the past 3 months and 37% of teen, ages 14-15, reported to having concurrent sexual partners [28].

#### Adherence to Self-Monitoring via Interactive Voice Response Targeting Weight Gain Prevention Among Black Women

A study was conducted by Steinberg et al to look at the adherence to self-monitoring via interactive voice response (IVR) technology in an eHealth intervention targeting Weight Gain Prevention among Black Women. The purpose of the study was to examine the correlation between IVR self-monitoring and weight change among black women of a lower SES enrolled in the Shape Program, which was an 18-month randomized controlled study comparing an eHealth weight gain prevention intervention with usual care among overweight and class 1 obese black female primary care patients. Intervention participants were asked to self-monitor their behavior and change goals via weekly IVR phone calls, which lasted 2 to 4 min on average. Other area of focus consisted of incorporating behavior change goals to promote weight change, tailored skills training, monthly training calls with a registered dietitian, and a 12-month gym membership. There were a total of 91 participants with a total IVR completion of 72% over the 12 months. The rate of IVR completion had a positive correlation with weight loss; participants with an IVR completion of 80% showed higher weight loss numbers than participants with less than 80% completion rate [29].

#### Mobile Phone Text Messaging Intervention for Cervical Cancer Screening

Because Korean American women have the highest mortality rates in regards to cervical cancer, Lee et al aimed to study how mobile phone text messaging can be used to promote cervical screenings and assess changes in knowledge and behavior. A focus group with 13 women from the target population was developed according to the community-based participatory research approach. The focus group determined the target populations’ actions, motivators, and barriers. This information was used to create a text campaign for 30 study participants that was tailored specifically to each participant’s time and educational needs. Participants received their personalized texts about cervical health and screenings at their specified times for 7 consecutive days. Comparing baseline data and post intervention data, there were statistically significant increases in knowledge of general cervical health, cancer, risk factors, and pap tests as well as change in beliefs about pap tests. Moreover, 23% of participants received pap tests, 83% of participants were satisfied with the program, and 97% stated that they would recommend the program to others [30].

#### Text Messaging to Motivate Exercise Among Latino Adults at Risk for Vascular Disease

This study developed a 2-part study with a questionnaire and 6-week trial first, distributing a 15-item survey to determine how often Latino adults use their phone. The second part of the study focused on exercise. Potential participants were to be 50 years or older with one or more atherosclerotic risk factors. The trial consisted of a questionnaire to measure physical activity and time active. Participants then received 30 text messages using software that allowed confirmation receipts of text messages. Authors found that mobile phone use and texting is common in the Latino population and is a viable option to motivate physical activity among this target group [31].

#### Automated Pain Intervention for Underserved Minority Women With Breast Cancer

This study focuses on high-risk underserved minorities suffering from cancer. The study examined IVR in assessing patients’ pain level and barriers to controlling pain, which accessed (1) patient pain levels and symptoms, (2) determination of pain and symptoms that exceed a threshold of severity, and (3) feedback about symptoms for physicians. When barriers were reported, staff would contact the patient to provide them with educational material. The study consisted of 60 low-income African American and Latina women who had breast cancer and cancer-related pain. Participants in the intervention group received phone calls twice a week, whereas the control group received usual care of pain and symptom management given by the clinic. Results showed that patients enrolled in the intervention group had a significant decrease in the proportion of women reporting moderate to severe pain. There was a decrease in the control group as well, but it was not statistically significant. The IVR intervention improved other cancer-related symptoms such as sleep disturbance and drowsiness [32].

#### Mobile Surveillance for Acute Respiratory Infections and Influenza-Like Illness in the Community

Stockwell et al used mobile surveillance for reported acute respiratory infections and influenza-like illness in the community. Data were collected for 44 weeks from 789 people, making up 161 households, in a community that primarily classified themselves as Latino. They received SMS text messages twice a week, urging them to report if anyone in the household was sick. The designated reporter of the household received the following SMS text message: Reply with 1 or 2. Does anyone in the household have runny nose, congestion, sore throat, cough, body aches, or fever, or feels [sick] hot? 1: yes; 2: no. If participants reported they were ill, a home visit was then conducted to obtain a nasal swab. Overall, 11,282 SMS text messages were sent, with a 73% response rate in the first month. From the time a patient showed symptoms, it took a median of 2 days for them to receive a nasal swab. Results of the samples obtained showed 236 samples (65%) were positive for a respiratory pathogen and 44 (19%) were positive for influenza. The nasal swabs also detected 12 other pathogens including coronavirus and respiratory syncytial virus. The use of SMS text messaging for interventions allows participants to report symptoms in a timely manner and also allows for a large number of participants to be simultaneously monitored [33].

## Discussion

### Principal Findings

Mobile technologies used to access and distribute health information have great potential to ameliorate outcomes within health care in the United States. This is imperative as it relates to underserved populations, as they suffer from the poorest of health outcomes, comprise the population served most via government assistance programs such as Medicaid and Medicare, and contribute to the astronomical health care cost and expenditures to our health care system. As Kumar et al explains, these technologies can support continuous health monitoring at both the individual and population level, encourage healthy behaviors to prevent or reduce health problems, support chronic disease self-management, enhance provider knowledge, reduce the number of health care visits, and provide personalized, localized, and on-demand interventions in ways previously unimaginable [34]. This is important when we compare the potential of mHealth in underserved, low SES population groups where traditional mass media health campaigns have been unsuccessful. Messages need to be designed to meet the “literacy, language, cultural and motivational needs of the population [35]. mHealth technologies can meet these needs by effectively tailoring health information using a tool that most patients already use or have access too. Due to this, mHealth has proven to be a potential route in reducing the incidence and prevalence of health disparities among our disadvantaged and underserved populations.

Although this systematic literature review revealed a number of mHealth interventions targeting underserved and minority populations here in the United States from a public health perspective, there are still several health problems that would make logical targets for SMS and mHealth interventions. Most of the current apps are developed from the perspective of the health care system for the general population versus public health interventions to underserved communities. As a result, research regarding the use of mHealth interventions for the populations that need it the most remains sparse. All chronic health conditions experienced by underserved populations could benefit from mHealth interventions due to the vast range of apps they can cover. As Nundy et al argues, wider mobile phones usage among minority groups has expanded access to address health disparities [36]. However, though the numbers of mHealth pilot studies are increasing, many of these pilot studies are focused solely on the assessment of mobile phone usage and ownership and fail to follow through with an implementation component to the study design.

One of the most notable mHealth interventions in the United States was the text4baby program dedicated to improving maternal and child health outcomes among low-income, minority, and underserved women [17]. The program utilizes culturally tailored health messages and uses in depth strategies to survey and identify the optimal methods for delivery of service and care among the target population. It also includes regular evaluation to ensure that the frequency, length, and content of the messages sent to participants are appropriate. Additionally, the literacy levels of the population that the text4baby program serves is always taken into consideration in the development phase [17].

### Evaluation Strategies and Theoretical Models

Despite the promise, some key challenges remain. As mHealth is still a relatively new field, different methods used and standards for measuring success varied from study to study based on the strategy used. This can affect the ability to compare results and implications across studies. Additionally, 7 of the studies in this review were randomized control trials and 3 were pilot studies that provided little to no information concerning the long-term effectiveness of mHealth strategies relating to that topic area.

Again, only 7 of the studies reviewed in this systematic literature review had theoretical constructs or grounding that guided the methods employed, and only 4 of those studies incorporated theories from health education and behavior, identified as Social Cognitive Theory (SCT), Transtheoretical Model, and the Health Belief Model [23]. This highlights the need for newer health behavior models applicable to mHealth interventions. As Evans et al argues, recent studies have noted both the relevance of existing behavioral theory, such as Social Cognitive Theory (SCT) and Theory of Planned Behavior (TPB), need to be examined and new models considered for their applicability to mHealth [23].

### Limitations

The review is limited by the nature of the literature search, which only assessed actual mHealth interventions among minority and underserved populations. An overview of the field indicates mHealth is becoming more common in health care and public health, and this collection of 16 articles does not represent the full range of projects being implemented using mobile phones in the United States. Interventions included in our review had to outline in the title, abstract, or in the study’s participants sections that the target participants were members of minority or underserved population subgroups, thus severely limiting studies that could be examined. Names of specific race and ethnic groups (African American or black, Latino or Hispanic, Native American, Asian, etc) were not used during the search results, although doing so might have yielded more studies. We wanted to search on a broader more general level instead of focusing on a specific race and ethnicity. It may be beneficial to conduct searches for specific races and ethnicities. Additionally, because the term mobile health has been previously associated with mobile health units, which are also used to increase access to care in low-income and disparate populations, it was decided to limit articles to those published between 2009 and 2016, which could have eliminated mHealth studies published earlier. Finally, when completing the literature search using key search phrases in the institution’s e-library database, 3 of the search phrases returned more than 2000+ articles; however, articles past the first 1000 articles could not be accessed. Therefore, for those 3 key phrases, search filters had to be applied individually rather than all at once, which could have affected the overall number of articles obtained

### Conclusions

mHealth has potential for influencing behaviors within public health and health education, particularly with regard to underserved and minority populations due to increased access to smartphones. Although projects using SMS text messaging interventions in low-income populations globally have begun to develop a strong evidence base for success, more research studies need to be conducted in the United States using SMS text messaging and additional mHealth strategies such as mobile apps gamification, and mobile Web (mWeb). In addition, more dissemination and implementation studies need to be conducted. Several pilot studies have been conducted, but it is important to understand and explore this research in the real world with different populations.

Although many of the studies reviewed in this systematic literature review primarily applied to populations in urban and metropolitan areas, those in rural populations can also benefit from mHealth technology. According to the Information and Communication Technology Policy Division of the World Bank, with every 3 out of 4 people living in developing countries or from low socioeconomic populations, the value and benefits of mobile phone services are considerably higher in rural areas [37]. According to PEW Research Center, Mobile technology use among rural adults has also risen rapidly, with the share of those owning smartphones and tablets increasing sharply. Ownership of desktop or laptop computers, in contrast, has only slightly risen since 2008.

In the United States, statistics indicate that during 2011 and 2012, there was a 13% increase in smartphone ownership among rural populations. As of 2014, we see that among young adults, 52% of rural populations compared with urban (68%) and suburban (66%) own smartphones, indicating a decreased gap [38]. Increased technology use among rural populations increased the opportunity for easier, faster, and more efficient access to health information for this population subgroup. As Bhavnani et al explains, mobile services are being used to disseminate locally-generated and locally-relevant educational and health information, in order to target rural communities whose populations typically have low levels of education and income and would not otherwise benefit from such information. There is evidence to suggest that this type of benefit could save lives in rural communities [38]. This highlights the need for similar mHealth interventions to benefit this population subgroup.

## Appendix

Multimedia Appendix 1 Research study background.

## Abbreviations

IVR: interactive voice response
mHealth: mobile health
PRISMA: Preferred Reporting Items for Systematic Reviews and Meta-Analyses
SCT: social cognitive theory
SES: socioeconomic status
SMS: short message service
STI: sexually transmitted infection
