# Supplementary material for: mHealth Technology Use and Implications in Historically Underserved and Minority Populations in the United States: Systematic Literature Review
Source: JMIR Mhealth Uhealth. 2018 Jun 18;6(6):e128. doi: 10.2196/mhealth.8383 (PMC6028762; doi:10.2196/mhealth.8383)
Supplement: Multimedia Appendix 1 [file mhealth_v6i6e128_app1.pdf]

Research Study Background								
**Author**	**Journal & Year of Publication**	**Population**	**Location**	**Health Focus**	**Study Design**	**Theoretical Grounding**	**Technology**	**Study Outcomes**
Fortune, T. et al.	Contemporary Clinical trials (2010)	African American Men (16 – 20 years old) who were sexually active.	Philadelphia, PA	HIV/AIDS prevention.	Non – randomized two group Pilot Study	N/A	Participant owned mobile phones.	Documented a 77% retention rate at 3 months and 65% retention rate at 6 months in their technology based HIV prevention initiative.
Katz, K.S et al.	Maternal Child Health Journal (2011)	African American/ Black and Latina primiparous pregnant women (15 – 19 years old)	Washington, DC	Interpregnancy Interval	Randomized controlled trial	A theoretical schema developed based upon social and youth asset development	Contracted mobile phones – 450 minutes per month, no texting, or internet features	GirlTalk intervention was not successful in reducing the time to subsequent pregnancy across the 24-month follow-up interval. 31% of the intervention group and 36% of the usual care group became pregnant again within 24 months. Among teen mothers aged 15–17 years, the rate of subsequent pregnancy was 26% in the

Research Study Background								
**Author**	**Journal & Year of Publication**	**Population**	**Location**	**Health Focus**	**Study Design**	**Theoretical Grounding**	**Technology**	**Study Outcomes**
								intervention and 39% in the usual care group. However, increased participation in the GirlTalk counseling program was associated with postponing a second pregnancy among teen mothers ages 15–17 years, but not those 18 and old.
Evans, W.D, Wallace, J.L., & Snider, J.	BMC Public Health (2012)	Low income Hispanic Women <20 – 35+ years of age	Farifax County, Virginia	Pregnancy	Randomized pilot study	Social Cognitive, Transtheoretical and Health Belief Models	Treatment group received prenatal counseling and SMS text messages for duration of pregnancy/	Mothers in the text4baby treatment group were nearly three times (OR = 2.73) more likely to believe that they were prepared to be new mothers compared to those in the no exposure control group.

Research Study Background								
**Author**	**Journal & Year of Publication**	**Population**	**Location**	**Health Focus**	**Study Design**	**Theoretical Grounding**	**Technology**	**Study Outcomes**
							program.	
Stockwell, M.S et al.	Journal of the American Medical Association (2012)	Parents of Latino Children (6 months – 18 years old)	New York, NY	Influenza and acute respiratory infection surveillance	Randomized controlled trial	N/A	Personalized SMS text messages sent in the language preferred indicated on the child's EHR.	When assessing the 3266 children and adolescents with presumed delivered text messages (86%), as of March 31, 2011, 47% of the intervention group (received text messages) vs 40% of the usual care group were vaccinated (*P*_.001). At the cohort-based fall review date, 29% of the intervention group vs 23% of the usual care group were

Research Study Background								
Author	Journal & Year of Publication	Population	Location	Health Focus	Study Design	Theoretical Grounding	Technology	Study Outcomes
								vaccinated (*P*_.001).
Gazmararian J.A. et al.	Maternal & Child Health Journal (2014	Black Women (18- 30 years old), SNAP/ WIC recipients and had a child < 10 months old or currently pregnant.	Atlanta, GA (Metro Atlanta)	Healthy Pregnancy Outcomes	Prospective Cohort Study	N/A	Mobile phone with ability to receive SMS text messages	Ninety-five percent of women reported receiving text4baby messages without interruption, 92% regularly read all messages throughout the study, and 88% planned to continue enrollment in the program.
Jones, R. & Lacroix, L.J.	AIDS Behavior (2012)	African American Women (18 – 29 years old) who were sexually active in the past 3 months	Northeast United States	HIV/AIDS and risky sexual behavior	Randomized Control Trial	Sex Script Theory and the Theory of Power as Knowing Participation in Change	Weekly video episodes streamed using Gmail accounts on smartphones provided.	At most, only 1 or 2 participants of the 117 in the video group missed an episode, and nearly all the participants watched each episode fully once or more than once, meaning they replayed a scene or re-watched the whole episode. he majority viewed the

Research Study Background								
**Author**	**Journal & Year of Publication**	**Population**	**Location**	**Health Focus**	**Study Design**	**Theoretical Grounding**	**Technology**	**Study Outcomes**
								videos on the smartphone at home (n = 97, 83%).
Joseph, R, Keller, C., Adams, M. and Ainsworth, B.	BMC Women's Health (2015)	African American women (24 – 29 years old)	Pheonix, AZ	Physical Activity	Randomized pilot study	Social Cognitive Theory	Call phones with ability to receive SMS text messages and access Facebook.	100% (n=14) of Facebook intervention (FI) group reported gaining physical activity knowledge from the culturally adapted promotion materials, were more motivated (94%; n=13), and saw messages as very helpful (79%; n=11) as compared to the print (PI) only group. The FI group reported significant pre-post intervention

Research Study Background								
**Author**	**Journal & Year of Publication**	**Population**	**Location**	**Health Focus**	**Study Design**	**Theoretical Grounding**	**Technology**	**Study Outcomes**
								enhancement in self-regulation for physical activity (p<.001) and social support for physical activity (p=.030).
DiClemente, R. et al.	JAMA Pediatrics (2014)	African American adolescent and young adults (14 – 20 years old) who reported having unprotected sex in the last 6 months	Atlanta, GA	STI and HIV/AIDS prevention	2 arm Randomized Trial	N/A	Mobile phone access required for telephone counseling.	For condom-protected sexual acts in both the 6 months and 90 days prior to assessment, significant differences were observed between conditions, with participants in the experimental condition (HORIZONS program + telephone counseling) reporting a significantly higher proportion of condom-protected sexual acts (P = .008 for the prior

Research Study Background								
**Author**	**Journal & Year of Publication**	**Population**	**Location**	**Health Focus**	**Study Design**	**Theoretical Grounding**	**Technology**	**Study Outcomes**
								90 days and P = .02 for the prior 6 months), fewer episodes of sex while high on drugs and/or alcohol in the prior 90 days (P = .01), and fewer vaginal sex partners in the prior 6 months (P = .046).
Nundy, S., et al.	Diabetes Education	African Americans aged 19 and older who were diagnosed with Type I & II Diabetes	Chicago, IL	Diabetes Self-Management	Fixed Method Observational Cohort	Behavioral Change Theory	Patients received SMS text messages about Diabetes Care	N/A
Khanna et al	Journal of Diabetes Science and Technology	Hispanic Men and Women (mean age =	Oakland, CA	Nutrition for improved	Randomized open-label with blinded	N/A	Mobile phone.	Despite a successful pilot study in Mexico there were no statistically or clinically significant differences

Research Study Background								
**Author**	**Journal & Year of Publication**	**Population**	**Location**	**Health Focus**	**Study Design**	**Theoretical Grounding**	**Technology**	**Study Outcomes**
		52 years old)		glycemic index and waist circumference	endpoint assessment			between these 2 groups in changes in A1c (–0.3% in the control group, –0.1% in the intervention group), or in any other anthropometric or laboratory measure.
Devine, S., Bull, S., Dreisbach, S. and Shlay, J.	Journal of Adolescent Health	Minority youth under the age of 20	Denver, CO	Reducing Teenage Pregnancy and School Dropout	Pilot study	Theoretical Model	Mobile phone with ability to receive SMS text messages	Analysis indicated a 49% response rate to messages that requested a response and a decrease in response rate over time. Qualitative data indicated that no more than on text per day was ideals, as 15% of younger teens would not have access to a mobile phone with texting capabilities therefore requiring them to buddy up with an older teen.

Research Study Background								
**Author**	**Journal & Year of Publication**	**Population**	**Location**	**Health Focus**	**Study Design**	**Theoretical Grounding**	**Technology**	**Study Outcomes**
Steinberg, D. et al.	Journal of Medical Internet Research	Black women (25 – 44 years of age)	North Carolina	Weight Control	Randomized control trial	N/A	Mobile phone with ability to receive SMS text messages	At 12 months, IVR call completion was significantly correlated with weight loss (r =-.22; P=.04) and participants with an IVR completion rate ≥80% had significantly greater weight loss compared to those with an IVR completion rate <80% (-1.97 kg, SE 0.67 vs 0.48 kg, SE 0.69; P=.01). Similar outcomes were found for change in body mass index (BMI; mean difference -0.94 kg, 95% CI -1.64 to -0.24; P=.009). Older, more educated participants were more likely to achieve high IVR

Research Study Background								
**Author**	**Journal & Year of Publication**	**Population**	**Location**	**Health Focus**	**Study Design**	**Theoretical Grounding**	**Technology**	**Study Outcomes**
								call completion. Participants reported positive attitudes toward IVR self-monitoring.
Lee, H.Y. Koopmeiners, J.S. Rhee, T.G. Raveis, V.H. Ahluwalia, J.S.	Journal of Medical Internet Research (2014)	Korean American Women (21 – 29 years of age)	Minnesota	Cancer Screening/ Prevention	Quasi-experimental research design	Fogg's Behavior Model	Mobile phone access required.	Significant improvements were observed for general knowledge about cervical cancer (*P*<.001), Pap test knowledge (*P*<.001), Pap test attitudes and beliefs (*P*=.006), and knowledge about risk factors of cervical cancer and its screening (*P*<.001). There was also a significant reduction in socio-cultural barriers to cervical cancer screening (*P*=.001). Additionally, one study participant (3%, 1/30)

Research Study Background								
**Author**	**Journal & Year of Publication**	**Population**	**Location**	**Health Focus**	**Study Design**	**Theoretical Grounding**	**Technology**	**Study Outcomes**
								reported receiving the Pap test within 1 week after completing the mScreening program and 6 additional participants (20%, 6/30) reported receiving the Pap test by the 3-month follow-up visit, which represents a 23% (7/30) improvement in the proportion of participants receiving the Pap test (95% CI 10% to 42%) compared to baseline.
Collins, T. et al.	Preventing Chronic Disease: Public Health Research, Practice and Policy	Latino Men and Women (50 and older)	Wichita, KS	Physical Activity and Exercise Behaviors	2 part: survey & 6 wk. trial intervention	Stages of Change	Mobile phone with ability to receive SMS text messages	At baseline, the mean minutes per week of exercise was 55.91 (SD, 55.76), which increased to 201.82 (SD, 61.61) minutes per week at 6 weeks ($p<.003$) after receiving

Research Study Background								
**Author**	**Journal & Year of Publication**	**Population**	**Location**	**Health Focus**	**Study Design**	**Theoretical Grounding**	**Technology**	**Study Outcomes**
								PACE text messages.
Anderson, K. O et al.	Journal of Breast Cancer Pain Intervention	African American and Latino Women 18 years of age and older	Houston, TX	Pain management in those with breast cancer.	Pilot study	N/A	Automated, telephone – based interactive voice response (IVR) system	During the study, 71% of the IVR assessments were completed successfully. during the study, 28 of the 31 patients in the intervention group reported symptoms severe enough to generate an alert, and 100% of the symptom threshold events were detected. E-mail alerts were sent to the physicians, who acknowledged receipt of 161 (73%) via e-mail response to the research staff.
Stockwell, M.S. et al.	American Journal of Epidemiology	Latina Men and Women (all ages) that	New York, NY	Surveillance	Community Based	N/A	Mobile phone with ability to	Response rates remained high throughout the study period (range, 68%–84%

Research Study Background								
**Author**	**Journal & Year of Publication**	**Population**	**Location**	**Health Focus**	**Study Design**	**Theoretical Grounding**	**Technology**	**Study Outcomes**
	(2014)	has households of 3 or more members		of Influenza like illnesses (ILI) and Severe acute respiratory infections (SARI)	Study		receive SMS text messages	per month). One-quarter (24.9%) of the swabs collected were obtained from children under 5 years of age; their positivity rate was 72%, and 8 (12%) were positive for influenza.
